# Computational Learning Model for Prediction of Heart Disease Using Machine Learning Based on a New Regularizer

**DOI:** 10.1155/2021/8628335

**Published:** 2021-11-11

**Authors:** Abdulaziz Albahr, Marwan Albahar, Mohammed Thanoon, Muhammad Binsawad

**Affiliations:** ^1^College of Applied Medical Sciences, King Saud Bin Abdulaziz University for Health Sciences, Al-Ahsa 31982, Saudi Arabia; ^2^Department of Science, Umm Al Qura University, P.O. Box 715, Mecca, Saudi Arabia; ^3^King Abdulaziz University, Computer Information System Department, Jeddah, Saudi Arabia

## Abstract

Heart diseases are characterized as heterogeneous diseases comprising multiple subtypes. Early diagnosis and prognosis of heart disease are essential to facilitate the clinical management of patients. In this research, a new computational model for predicting early heart disease is proposed. The predictive model is embedded in a new regularization based on decaying the weights according to the weight matrices' standard deviation and comparing the results against its parents (RSD-ANN). The performance of RSD-ANN is far better than that of the existing methods. Based on our experiments, the average validation accuracy computed was 96.30% using either the tenfold cross-validation or holdout method.

## 1. Introduction

Cardiovascular disease, or CVD, refers to various heart disorders, including structural heart abnormalities and blood vessel blockages. Over 17 million individuals have died from cardiovascular disease, according to the World Heart Federation (WHF). Additionally, the World Health Organization (WHO) reports that cardiac disease has a greater yearly fatality rate than any other disease. In various domains of the biomedical sector [1–3], machine learning techniques have been successfully utilized. Machine learning has transformed the biomedical industry, paving the way for several new methods for simplifying the identification of various disease classes. Applied mathematicians have long recognized the “curse of dimensionality” as a significant barrier to using complex biomedical data. Such data is distinguished by having a higher-dimensional space with fewer points. Reducing the feature space will help the classification models be more effective [4–5]. Once the irrelevant features are eliminated, efficient classifications and predictions of various diseases are possible [6–9]. Choosing an appropriate data preprocessing strategy is unavoidable, but classifying and predicting models still poses another challenge. It is highly difficult to diagnose cardiovascular disease, and we must do it with great accuracy and proficiency. Usually, the diagnosis is based on just assumptions and the doctor's experience. However, just like humans, we are not infallible, and our decisions could result in either a life-saving or a tragic outcome. In these critical situations, machine learning techniques have emerged. Instead of using human knowledge, this method can accurately foretell a disease. Selecting the correct categorization and prediction model is still considered a difficult task by many researchers. Without classifying and predicting CVD, these models' performance in classifying and predicting cardiovascular disease declines significantly. After developing such a model, the proper evaluation must be validated through a strong channel, such as using a medical and research institute for heart disease detection and prediction. This study proposed a new predictive model for early heart disease detection, in which weight decay is used to shrink the influence of each data point in the weight matrix. Once weight decay has been applied, the influence of each data point is multiplied by *λ* to obtain the regularization term. To summarize, this paper has contributed the following to the literature.

We compare the performance of RSD-ANN with other models. Our experimental results indicate that our model achieved relatively higher results than those of previously published methods. We present the design and implementation of a regularizer based on the Relative Standard Deviation for the Artificial Neural Network (RSD-ANN) system. Furthermore, we study the performance of RSD-ANN in combination with the PCA transform and different existing regularizers with varying parameters of regularization and their impacts on the accuracy.

This paper is organized as follows: Section 2 discusses the methods employed in the detection of heart diseases.  Section 3 explains the use of the dataset for heart detection. In Section 4, the benefits of the regularization methods are discussed. In Section 5, the proposed architecture is discussed.  Section 6 explains the training and validation process.  Section 7 discusses the efficiency of the architecture through evaluation matrices and provides a comparison with the state-of-the-art methods. Finally, conclusions are drawn, and future directions are discussed in Section 8.

## 2. Related Work

For complex parameter interpretation across multiple categories of data, machine learning methods outperform statistical approaches in both accuracy and precision. Machine learning techniques produce accurate and robust predictions based on a small set of assumptions [10], and they are becoming increasingly popular. Machine learning techniques are either classified as unsupervised learning methods or supervised learning methods. A trained dataset is not required for the former, but it is required for the latter. Gavhane et al. [11] devised a model for analyzing and monitoring the human cardiovascular system. This model was created to detect coronary artery disease in patients. They used the Cleveland dataset. The dataset has 76 properties and 303 instances. The authors used 13 of the 76 available attributes in the analysis process. The model detected coronary artery disease using Bayes networks, Functional Trees (FT), and Support Vector Machines (SVM). SVM-based holdout tests were 83.8% accurate, whereas FT-based holdout tests were only 81.5%. The relevant attributes were selected using the Best First method. The accuracy of the FT was 84.5%, the Bayes net was 84.5%, and the SVM was 85.1%. Repaka et al. [12] used Naive Bayes to design a method for detecting cardiac disease. Due to its application of the Bayes Theorem, this strategy became one of the most influential categorization models. WEKA was used to implement the solution. The model had an accuracy of 86.419%. However, for larger datasets, the model's reliance on the Bayes Theorem cannot be validated. Babu et al. [13] proposed several methods for classifying cardiovascular diseases. This solution was implemented using the WEKA tool. Machine learning techniques such as Bagging, Naive Bayes, and J48 were employed. The J48 technique had an accuracy of 84.35%. The naive Bayes algorithm was 82.31%, while the Bagging strategy was 85.03%, making it the more accurate method of the three. Parthiban and Srivatsa created a predictive model for diabetic patients' CVD using Naive Bayes and SVM [14]. Only 142 people out of 500 were impacted, while the remaining patients were unaffected by the condition. The Naive Bayes algorithm was accurate to 74%. Tan et al. [15] proposed a hybrid approach using wrapper-based feature selection. It included Support Vector Machines (SVM) and Genetic Algorithm (GA) techniques. To analyze the proposed approach, WEKA and LIBSVM were employed. The authors used five datasets from the UC Irvine machine learning repository. The hybrid technique was applied to the heart disease dataset to achieve the highest accuracy of 84.07%. Ordonez [16] proposed a rule-based association method for heart disease prediction. The rule set was optimized using an algorithm. This algorithm discovered association rules in the training data and validated them on a separate test dataset. The significance of the associated rules in the medical field was validated using support and confidence values. This strategy resulted in the development of rules with a high degree of predictive accuracy for heart disease. Rairikar et al. [17] proposed a method for cardiac disease prediction. This system was developed using thirteen clinical characteristics extracted from a UC heart disease dataset. Algorithms based on the Learning Vector Quantization (LVQ) technique and Artificial Neural Networks (ANNs) were used to diagnose heart disease. The proposed system achieved a level of accuracy of approximately 80%. Nahiduzzaman et al. [18] developed a framework for diagnosing heart disease using SVM and Multilayer Perceptron (MLP). This method achieved 80.41%. Finally, Nahar et al. [19] proposed a novel hybrid CVD prediction technique that combines Computerized Feature Selection (CFS) and Medical Feature Selection (MFS). However, in the absence of a more precise classification methodology, this strategy resulted in an inefficient model. A multistage Convolutional Neural Network (CNN) model for diagnosing coronary heart disease was developed by Dutta et al. [20]. For data imbalance, this model proved to be resilient. However, lasso regression was used in this case because it assumes a linear relationship between the input factors and the labels. Worth noting, unbalanced data lead to incorrect classification of data. Tougui et al. proposed a model for classifying and predicting heart disease using ANN and SVM in [21]. The authors achieved an accuracy of 84.7%. In addition, they utilized 12 risk factors, such as cholesterol, food, age, sex, and blood pressure, to develop a genetic neural network algorithm for predicting heart diseases. 50 individuals from the American Heart Association volunteered to take part in the study. Though this approach has some merit, it also has a couple of drawbacks. The allocation of neural networks is a random process, and the neural network's performance is adversely affected when searching for the optimal global value. The other disadvantage of adopting the backpropagation technique is that it may result in the neural network failing to achieve convergence. Pathak and Arul Valan [22] proposed predictive models for cardiac disease using trees, SVM, Naive Bayes, Neural Networks, and fuzzy approaches. The F-measure, accuracy, recall, and precision of the system were all tested and satisfactory. However, there was no suitable feature selection process used in this research. Hence, the J48 classifier model was shown to be the most successful among the numerous models tested in this study.

Baitharu and Pani [23] proposed a study primarily concerned with healthcare decision-making and would employ various techniques, including J48, IBK, VFI, Nave Bayes, Multilayer Perceptron, and Xero. The accuracy attained was extremely low, and it is, therefore, unsuitable for use in healthcare decision-making situations. Bouaziz et al. [24] presented a K-NN technique for predicting cardiac illness based on wavelet analysis. The primary goal was to detect cardiac disease with the smallest number of features possible. After the samples have been generated for each output class, the suggested Average KNN computes the Euclidean distance between them to quickly identify and pick the nearest neighbors. The method has lower accuracy and is less efficient than other methods. Sharma and Saxena [25] developed a genetic algorithm-based technique for predicting cardiac disease in their paper. The accuracy of this technique was 73.46%, based on the utilization of 14 different attributes. This approach is inefficient and unsuitable for decision-making. Using a hybrid model ensemble feature selection approach, Tripathi et al. [26] proposed a method for credit scoring. The efficacy of this methodology was purely dependent on the selection of the most appropriate classifiers for the ensemble. Using a rough set and multlayered ensemble for classification, Tripathi et al. [27] created another hybrid credit scoring model implemented in the real world. Both models are well suited for use in credit scoring situations. Balogun et al. [28] suggested a technique that used four-filter feature ranking (FFR) and a fourteen-filter feature subset selection to determine the best filter feature to use (FSS). This technique improved the predictability of the inducers and is particularly well suited for diagnosing software defects. Akintola et al. [29] investigated the impact of filter-based attribute selection approaches on predicting software defects. The ten datasets from the NASA and Metric Data Program software repository were evaluated using the Principal Component Analysis (PCA), CFS, and Filter Subset Evaluation methods developed by the researchers. Balogun et al. [30] investigated the effects of 46 feature selection strategies using Naive Bayes and decision tree classifiers to determine their effectiveness. The use of probability-based, statistical-based, and classifier-based FFR approaches was advocated in this review. This approach is particularly well suited for discovering software flaws. Kolukisa et al. [31] established a novel adaptive and optimized ensemble technique to analyze coronary artery disease (CAD) that is both fast and accurate. Even though this strategy employed an ensemble approach for classification, it did not use any pretreatment techniques before classification.

On the other hand, Latha and Jeeva [32] looked at numerous ways of enhancing the efficiency of weak prediction approaches. When it came to classification, the strategy used both bagging and boosting ensembles. As described by conducting a survey of various CVD prediction techniques, the dimensionality of a medical dataset is extremely high, necessitating the use of an efficient feature selection mechanism to keep the number of attributes to a minimum while also incorporating an efficient machine learning model to improve prediction accuracy. Additionally, there is a lack of suitable classification and prediction methodologies for accurately classifying and forecasting data on heart disease. Additionally, with recent advancements in classification techniques such as ensemble learning, there may be a way to improve prediction accuracy and efficiency in the future. This study established a new predictive model for identifying heart disease diagnosis in patients using a new regularizer. RSD-ANN has several advantages over other models, including requiring less computing time and providing higher generalization capabilities.

## 3. Dataset

The heart disease dataset used in the experiments is available on the University of California's open repository. There are 303 instances, 76 features, and 2 classes in total (absence and presence of heart disease). Each instance contains information about a patient's heart disease diagnosis and physical and biochemical constants commonly used in medical diagnosis. Notably, this is one of the most commonly used open datasets in medical machine learning papers [33]. We only used 14 features for this study, including the class attribute, which has the following distribution: the absent class had 150 instances, corresponding to 55.5% of the dataset, and the present class has 120 instances, corresponding to 44.5% of the dataset. As shown in Table 1, the class values are binary: they answer “yes” or “no” to the question of existing heart disease.

**Figure 1:**
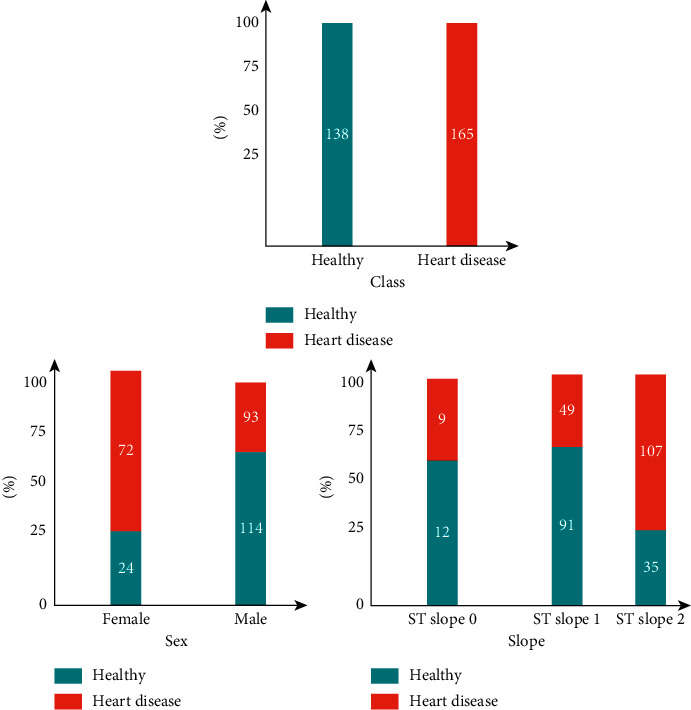
Categorical features of mosaic plots.

**Figure 2:**
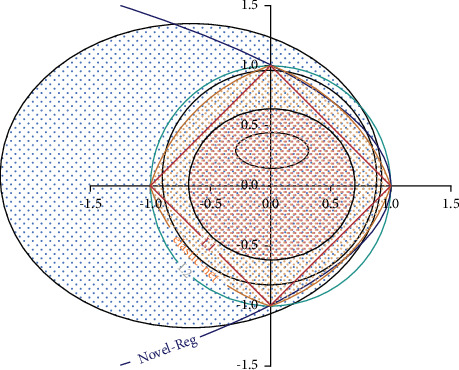
Contours representation of L1, L2, elastic net, and new regularizers.

Statlog's categorical features are depicted in Figure 1 as a mosaic plot. The horizontal axis displays the values for categorical or discrete attributes, with the number representing the number of people who have that characteristic value. The proportion of people with and without heart disease for a particular value of a characteristic is called the height proportion. There appeared to be a specific link between some characteristics and heart disease. For instance, when the slope was type 2, the risk was more significant for women than for males, and there was a significant association with heart disease. Differentiating the values according to the density function illustrates the association between numerical and cardiac diseases (Figure 2). The plots indicate that there may be a correlation between age, the maximum heart rate achieved during an exercise test (thalach), and the depression generated by activity at rest (oldpeak) in individuals with or without cardiac disease.

## 4. Regularization: Control the Model Complexity

As a predictive model is trained, the learning model may begin to memorize the data, causing the generalization error to increase. As a result, the model performs admirably on training data but dramatically worsens on unknown or test data. The process of avoiding memorization is known as “regularization.” The goal of regularization is to penalize the learning model for starting to generalize by performing well on previously unseen data. Penalty terms are imposed on the prediction model by various types of regularizers. The most commonly used regularization methods include L1 and L2 regularizers. A more in-depth discussion of the L1 and L2 functions, as well as the elastic net and new regularizers, is provided below.

### 4.1. Lasso Regularizer

L1 or lasso regression is a type of regression model that employs L1 regularization in the context of regression modeling. The L1 regularization multiplies the coefficient by the sum of the absolute values of the coefficients, which is the most straightforward kind of regularization. This assists us in deciding which features to include in our model since it downsizes the less critical ones while removing the less important ones (making them zero). In mathematical language, lasso regression penalizes the loss function by including the absolute value of the coefficient of the regression model as a penalty term [34]:

(1) $$\begin{array}{c} \sum_{\dot{I}=1}^{n}{\left(y_{i}-\sum_{j=1}^{p}x_{ij}\beta_{j}\right)}^{2}+\lambda \sum_{j=1}^{p}|\beta_{j}, \end{array}$$

where *P* is the total number of features in the data, *β* is the weight values associated with each feature, and *j* is the *j* − th row of the weight matrix. *λ* is the model's regularization parameter. Reduced overfitting is achieved by increasing *λ*. The regularization term multiples by *λ* (scalar), which modifies the overall effect of regularization. Consequently, increasing the *λ* value will improve the regularization impact.

### 4.2. Ridge Regularizer

The term “L2 or ridge regression” refers to L2 regularization. The parameters must be reduced to regularize the coefficients. The coefficients shrink in size when a penalty is applied. The additional penalty associated with ridge regularization is equal to the total of the squared values of the coefficients added to the loss function [34]:

(2) $$\begin{array}{c} \sum_{\dot{I}=1}^{n}{\left(y_{i}-\sum_{j=1}^{p}x_{ij}\beta_{j}\right)}^{2}+\lambda \sum_{j=1}^{p}\beta^{2}{}_{j}, \end{array}$$

where *P* is the weight matrix row for each feature, *β* is the feature's coefficient value, and *j* is the *j* − th row of the weight matrix. The correct value for *λ* must be found carefully. A large Lambda overfits the training data, leading to underfitting. In general, L2 regularization does an excellent job of reducing overfitting.

### 4.3. Elastic Net Regularizer

In the elastic net linear regression, regression models are regularized using both the lasso and ridge techniques. By combining the ridge and lasso techniques and learning from their shortcomings, the technique improves the regularization of statistical models. The elastic net approach addresses lasso's constraints, such as when only a few samples are required for high-dimensional data. The elastic net approach allows for the incorporation of “*n*” variables until saturation is reached. If the variables are highly correlated, lasso will usually pick one from each group and ignore the rest. To increase the versatility of the elastic net, a quadratic expression (||*β*||^2^) is added to the penalty (a type of ridge regression when applied in isolation) because the quadratic expression in the penalty elevates the loss function toward being convex. Elastic net is a hybrid of ridge regression and lasso regularization that excels at modeling data with many strongly correlated predictors. Consider a data matrix with dimensions of *p*, where *p* denotes the number of predictor variables and a solution vector with dimensions of *n*, where *n* denotes the number of observations. The goal of an elastic net is to minimize the following loss function:

(3) $$\begin{array}{c} \lambda \sum_{j=0}^{p}\frac{\left(1-a\right)}{2}\;\beta_{j}^{2}+a\beta_{j}, \end{array}$$

where *λ* is the regularization parameter and *α* is the mixing parameter. The *λ* parameter is nonnegative, that is, *λ* ∈  [0,  *∞*). When the value of *λ* is zero, the regularization has no effect. In other words, the only goal is to reduce the loss function to its smallest possible value. As the value approaches infinity, the regularization effect becomes more pronounced. Instead of minimizing the loss function, the only goal is to keep the coefficients *β* as small as possible. When *α* = 0, the elastic net is the same as ridge regression (i.e., a set of correlated predictors' coefficients are similarly reduced toward zero). In contrast, when *α* = 1, the elastic net is the same as the lasso regression (one of the correlated predictors has a larger coefficient, while the rest are shrunk to zero).

### 4.4. New Regularizer

In general, the L1 regularizer selects or reduces features, whereas the L2 regularizer reduces the weights of unimportant features. The lasso fails to provide a grouped selection, which is the primary shortcoming of regularizers of this type of selection. It has a tendency to select one variable from a group while ignoring the rest in the group. Aside from that, the elastic net contributes to reducing the impact of specific features while not totally eradicating them. Furthermore, they manage individual weights without taking into account the relationship between the weight matrix entities. To address this constraint, we designed a new regularizer that considers the weight values' dispersion. This regularizer is referred to as a standard deviation-based regularizer (RSD). The new regularizer computes the regularization term by multiplying the weight matrix's standard deviation by *λ*. The goal is to build a more adjustable weight decline method. As a result, the regularizer restricts the learning model from utilizing global values from the weight space as input (see Figure 1).

As depicted in Figure 2, the new regularizer's outlines are presented in detail. Specifically, the penalty for all regularizers (L1, L2, elastic net, and the new) is equal to one in this case. We have also omitted the sum factor from the suggested regularizer to preserve its dimensions. As a result, the regularizer's spread depends on the penalty term *λ*. The spread expands as the penalty term *λ* is reduced, and the spread shrinks as the penalty term *λ* increases. The equivalent mathematical formulation for the new regularizer is provided as follows:

(4) $$\begin{array}{c} \sigma \left(w\right)=\frac{1}{P_{k}}\left\{\sum_{j=1}^{P_{k}}B_{j}^{2}-\frac{1}{P_{k}}{\left(\sum_{j=1}^{p_{k}}B_{j}\right)}^{2}\right\}, \end{array}$$

where *k* denotes the row numbers in the weight matrix and *j* is the weight matrix's row whereas *σ* denotes the weight values of standard deviation. The parameter *λ* controls the weight matrix values, and *P* is the columns number in each *j* − th row of the weight matrix (*P* depends on the number of features in the dataset). So, *P* is the size of the weight vector. Hence, we minimize the loss function with respect to *w* through the standard deviation of *w* to adopt the specific range values. The Nesterov ADAM optimizer was used to train the model, which included tanh activation functions. The model was trained over 100 epochs. A feed-forward network was used to classify the labeled data.

## 5. Model Architecture

**Figure 3:**
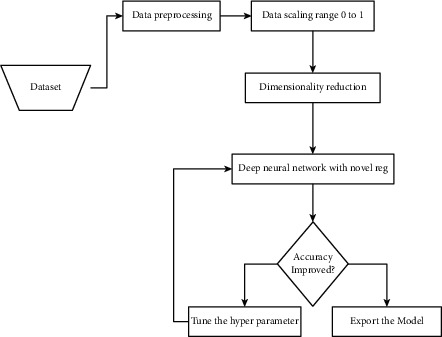
The general workflow of the proposed model.

To analyze the efficiency of RSD-ANN, we have performed certain steps given below. The functionality and dependency of each step are depicted in Figure 3.

1. Data preprocessing
2. Data scaling
3. Dimension reduction/feature selection
4. Classifier selection

### 5.1. Data Preprocessing

During data preprocessing, nominal and textual attributes are converted to numerical values through the label encoding algorithm. After that, the duplication of data is removed to avoid the classifier biased toward the majority class in data and thus affect the performance. Data preprocessing is a crucial task in both supervised and unsupervised learning.

### 5.2. Data Scaling

In the dataset, each attribute column has a different range of data. Some are continuous values having a low standard deviation, and others have a large range of values dispersed in feature space. To bring the data to equal mean and standard deviation, data scaling is performed. Through scaling, the information in the data is retained. In this step, both datasets were scaled according to the following equation:

(5) $$\begin{array}{c} X_{i}=\frac{X_{i}-\min\left(X_{i}\right)}{\max\left(X_{i}\right)-\min\left(X_{i}\right)}, \end{array}$$

for which *i* =  1,…, *k* where *k* represents the total number of rows and *X*_*i*_ shows the *i* − th feature in the data.

### 5.3. Feature Selection

Feature selection, also known as dimensionality reduction, completely removes unnecessary features from the data. For this purpose, we transformed the data into eight principal orthogonal components using a statistical method known as Principal Component Analysis (PCA) algorithm [35]. PCA was applied to the heart dataset, and all 13 features were reduced to eight correlated components.

### 5.4. Loss Function

To evaluate our classifier, we have used the cross-entropy loss function. The cross-entropy loss function is defined in equation (6). In neural networks, most of the time, the cross-entropy is given priority over other loss functions due to some specific reasons. For example, the squared loss function is suitable for regression. The other reason is that the output values of cross-entropy are between 0 and 1. Therefore, it is simple to convert the probabilistic values between 0 and 1 to either one class or another using different thresholds. Moreover, the cross-entropy loss function easily converges to the corresponding class values compared to other loss functions:

(6) $$\begin{array}{c} \text{Cross entropy}=\sum_{i}^{C}\left(t_{i}\log\;f\left(s_{i}\right)\right), \end{array}$$

where *C* is the number of classes, *t*_*i*_ is the *i*th class, and *f*(*s*_*i*_) is the *i*th output after activation function *f*.

## 6. Training and Validation Process

We chose Python as the implementation language for simulations, and the Keras framework is used for ANN. The ANN model is embedded with a new regularizer to test our method. Using the mathematical description given in equation (3), the proposed regularizer is implemented as a function. This new function was assigned to the kernel regularizer in the ANN model instead of the built-in regularizers. Two hidden layers of five and three units each make up the ANN predictive model. According to the class values, the last layer has two units. Except for the final layer, where the softmax activation function is used, each layer uses the tanh activation function. In the first two layers, the weight matrix was initialized with a Gaussian random distribution. For 100 epochs, the ANN model is trained on 80% of the data and validated on 20% of the data, with a batch size of 4 for each iteration. As a result, the results of multiple simulations were recorded and plotted. The following section contains the results and discussion.

## 7. Results and Comparison

### 7.1. Embedding L1 Regularizer with ANN

**Figure 4:**
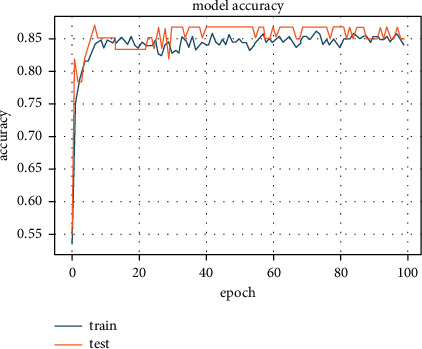
Accuracy of 85.14% achieved with L1 regularizer.

The default L1 regularizer was embedded with ANN learner and trained for different values of *λ*. This regularizer's best results were with *λ* = 10^−8^, and accuracy was 85.14% (see Figure 4).

**Figure 5:**
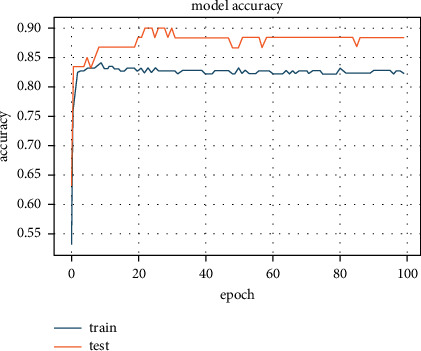
Accuracy of 87.7% achieved with L1 regularizer with PCA algorithm.

The L1 regularizer application, in combination with the PCA algorithm, increased the accuracy to 87.7%, which was the best accuracy possible (Figure 5). This increase in accuracy can be ascribed to the removal of less important features through PCA transformation. PCA only keeps the correlated features, which has a strong correlation with possible categories in data.

### 7.2. Embedding L2 Regularizer with ANN

**Figure 6:**
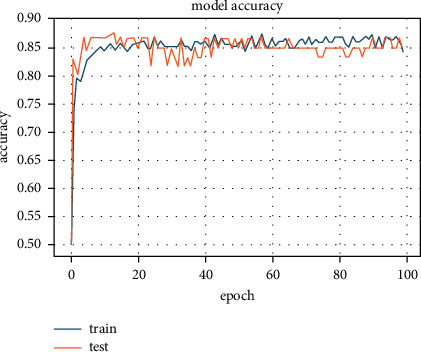
Accuracy of 84.8% achieved with L2 regularizer.

Similarly, in this step, we replaced the L1 regularizer with the L2 regularizer in the ANN model and searched for the best parameters to yield better accuracy. As a result, 84.8% accuracy was recorded with *λ* = 10^−9^ (see Figure 6).

**Figure 7:**
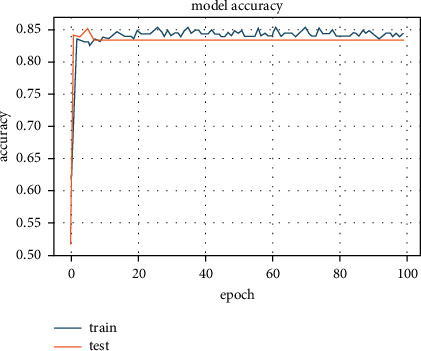
Accuracy of 83.04% achieved with L2 regularizer with PCA algorithm.

We observed a decrease in the average accuracy when combining the PCA transform with the L2 regularizer. The average accuracy for this stage was 83.04% (see Figure 7). This is because the L2 regularizer does not assign zero values to attribute coefficients. Hence, the less important features are also incorporated, due to which the accuracy decreased.

### 7.3. Embedding New Regularizer with ANN

**Figure 8:**
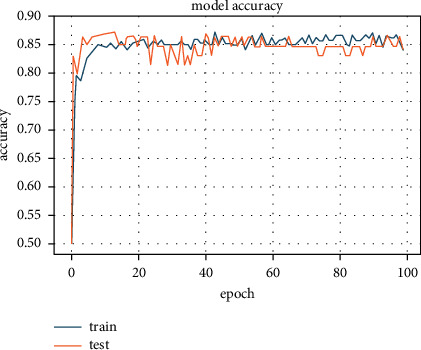
Accuracy of 88.59% achieved with new regularizer.

Based on our results' analysis, the new regularizer showed a significant improvement in terms of accuracy. The accuracy of our new regularizer outperformed L1 and L2 regularizers. The average accuracy obtained was 88.59% (see Figure 8).

**Figure 9:**
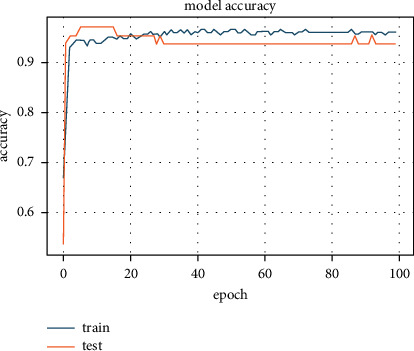
Accuracy of 96.30% achieved with new regularizer with PCA transformation.

**Figure 10:**
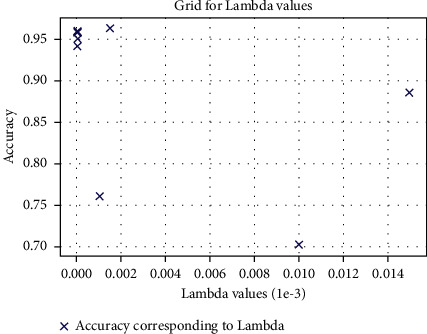
Lambda values and corresponding accuracy for each fold out of 10 folds.

We then combined the PCA algorithm with RSD-ANN to measure accuracy. As a result of this combination, the accuracy jumped to 96.30%; see Figure 9. During the PCA transformation, the number of features was reduced to eight orthogonal correlated components. As an important note, the accuracy improved because PCA projects each feature to maximum variance in feature space. The new regularizer controls the spread of the weight values in weight space (see Figure 10). Hence, the data becomes more separable for the ANN classifier, providing greater accuracy as a result. The same architecture was used for this simulation, and *λ* values were slightly increased to 10^−6^.

**Figure 11:**
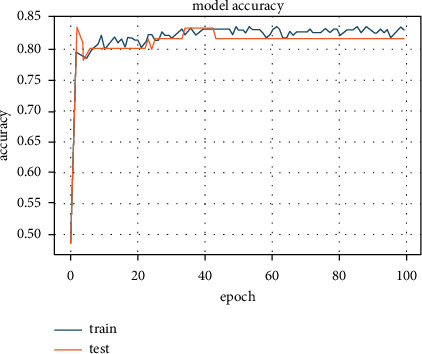
Accuracy achieved 81.08% for the combination of PCA with elastic net regularizer.

To validate our proposed model's effectiveness, we compared the RSD-ANN results with the results obtained from combining elastic net regularizer and PCA transform with eight components on the heart dataset. An accuracy of 81.08% was observed, which showed that the elastic net regularizer performed worse than the L1 and L2 regularizers separately. The accuracy plot for elastic net regularizer is shown in Figure 11.

Table 2 shows each embedded regularizer's experimental results with ANN, demonstrating that the new regularizer performed significantly better than the default regularizers. Therefore, the proposed regularizer is extensively effective.

We performed our simulations using RSD-ANN with a PCA algorithm. Out of 13 attributes, eight orthogonal correlative features were reproduced via PCA. The experimental outcomes of the proposed approach are compared to the results of other techniques currently available.  Table 3 shows the classification metrics (accuracy, sensitivity, specificity, and f-score) of RSD-ANN compared to other models. Based on our experimental results, it can be concluded that the RSD-ANN has outperformed all other models considered in the comparison. The GA-LDA + hybrid ensemble model achieved an accuracy of 93.65%. On the contrary, our model has the highest accuracy. Our model has achieved 93.75% in specificity, whereas the other approaches have rates below 90%. The sensitivity rate for the GA-LDA + hybrid ensemble is 96%, which is higher than the sensitivity rate of RSD-ANN due to identifying and selecting the best features for better prediction. It is worth noting that this measurement may cause issues if the data analyzed is affected by uncertainty or inaccuracies. Consequently, it limits its usability. However, by utilizing a fuzzy classifier, we can avoid these issues [36]. The experiment results demonstrate that our proposed method outperforms the other models in terms of accuracy in classifying cardiovascular disease. RSD-ANN was trained on a train-test split of 80–20% data. According to this division, the model was trained on 237 patients and tested on 60 patients.

## 8. Conclusion

In this paper, we present an efficient computational model for heart abnormality detection. We focused on a learning model that uses a new regularizer, which is purely based on the weight matrix's standard deviation. The new regularizer penalizes the coefficients of attributes from getting high values in the weight matrix space. The proposed model obtained excellent results, and it can be used to assist medical practitioners when searching for abnormalities in heart function. During the process, holdout and 10-fold validation were used, and the accuracy obtained for heart disease detection was 96.30%. Consequently, the incorporation of the proposed regularizer with ANN surpassed other methods in terms of accuracy.

## Figures and Tables

**Table 1:** Dataset description for heart disease.

S. no.	Attribute name	Value range
1	Age group	≥25
2	Gender	Male = 1, female = 0
3	Pain in chest	4 distinct values denote the intensity of pain
4	Diastolic pressure	≥94
5	Cholesterol quantity per mg/dl	≥126
6	Sugar quantity in blood per mg/dl	≥120
7	ECG values	0, 1 or 2
8	MHR	≥71
9	Angina induced	0 or 1
10	ST peak depression	≥0
11	ST segment elevation	1, 2 or 3
12	No. of great vessels considered	0, 1, 2 or 3
13	Thal value	Fixed defect = 6, reversible defect = 7, normal = 3.

**Table 2:** Dataset description for heart disease.

Regularization	Without PCA (%)	With PCA (%)
L1	85.14	87.5
L2	84.8	83.03
New	**88.59**	**96.30**

Best results are highlighted in bold.

**Table 3:** Proposed regularizer versus existing literature's results using 10-fold CV.

Reference	Method	Accuracy	Sen.	Spe.	*F*-score
[37]	BSWFM	87.4%	82.5%	91.3%	None
[38]	TWIST algorithm	84.14	74.23	78.87	None
[39]	ICA + SVM	83.75	80.67	79.28	None
[40]	GA-LDA + hybrid ensemble	93.65	96.00	89.25	None
	RSD-ANN	**96.30%**	**95.24%**	**93.75%**	**94.57%**

Best results are highlighted in bold.

## Data Availability

The datasets used to support the finding of this study are included within the article.

## References

[1] Antoniadis A., Lambert-Lacroix S., Leblanc F. (2003). Effective dimension reduction methods for tumor classification using gene expression data. *Bioinformatics*.

[2] Guyon I., Weston J., Barnhill S., Vapnik V. (2002). Gene selection for cancer classification using support vector machines. *Machine Learning*.

[3] Yu J. S., Ongarello S., Fiedler R. (2005). Ovarian cancer identification based on dimensionality reduction for high-throughput mass spectrometry data. *Bioinformatics*.

[4] Oh I.-S., Lee J.-S., Moon B.-R. (2004). Hybrid genetic algorithms for feature selection. *IEEE Transactions on Pattern Analysis and Machine Intelligence*.

[5] Remeseiro B., Bolon-Canedo V. (2019). A review of feature selection methods in medical applications. *Computers in Biology and Medicine*.

[6] Tallón-Ballesteros A. J., Correia L., Xue B. (2018). Featuring the attributes in supervised machine learning. *Lecture Notes in Computer Science*.

[7] Medina Garcia V. H., Rodriguez Rodriguez J., Ospina Usaquén M. A. (2018). A comparative study between feature selection algorithms. *Data Mining and Big Data*.

[8] Jain D., Singh V. (2018). Feature selection and classification systems for chronic disease prediction: a review. *Egyptian Informatics Journal*.

[9] Uyar K., İlhan A. (2017). Diagnosis of heart disease using genetic algorithm based trained recurrent fuzzy neural networks. *Procedia Computer Science*.

[10] Jordan M. I., Mitchell T. M. (2015). Machine learning: trends, perspectives, and prospects. *Science*.

[11] Gavhane A., Kokkula G., Pandya I., Devadkar K. Prediction of heart disease using machine learning.

[12] Repaka A. N., Ravikanti S. D., Franklin R. G. Design and implementing heart disease prediction using naives Bayesian.

[13] Babu S., Vivek E. M., Famina K. P. Heart disease diagnosis using data mining technique.

[14] Parthiban G., Srivatsa S. K. (2012). Applying machine learning methods in diagnosing heart disease for diabetic patients. *International Journal of Applied Information Systems*.

[15] Tan K. C., Teoh E. J., Yu Q., Goh K. C. (2009). A hybrid evolutionary algorithm for attribute selection in data mining. *Expert Systems with Applications*.

[16] Ordonez C. (2006). Association rule discovery with the train and test approach for heart disease prediction. *IEEE Transactions on Information Technology in Biomedicine*.

[17] Rairikar A., Kulkarni V., Sabale V., Kale H., Lamgunde A. Heart disease prediction using data mining techniques.

[18] Nahiduzzaman M., Nayeem M. J., Ahmed M. T., Zaman M. S. U. Prediction of heart disease using multi-layer perceptron neural network and support vector machine.

[19] Nahar J., Imam T., Tickle K. S., Chen Y.-P. P. (2013). Computational intelligence for heart disease diagnosis: a medical knowledge driven approach. *Expert Systems with Applications*.

[20] Dutta A., Batabyal T., Basu M., Acton S. T. (2020). An efficient convolutional neural network for coronary heart disease prediction. *Expert Systems with Applications*.

[21] Tougui I., Jilbab A., El Mhamdi J. (2020). Heart disease classification using data mining tools and machine learning techniques. *Health and Technology*.

[22] Pathak A. K., Arul Valan J. (2019). A predictive model for heart disease diagnosis using fuzzy logic and decision tree. *Advances in Intelligent Systems and Computing*.

[23] Baitharu T. R., Pani S. K. (2016). Analysis of data mining techniques for healthcare decision support system using liver disorder dataset. *Procedia Computer Science*.

[24] Bouaziz F., Boutana D., Oulhadj H. Diagnostic of ECG arrhythmia using wavelet analysis and K-nearest neighbor algorithm.

[25] Sharma P., Saxena K. (2017). Application of fuzzy logic and genetic algorithm in heart disease risk level prediction. *International Journal of System Assurance Engineering and Management*.

[26] Tripathi D., Edla D. R., Cheruku R., Kuppili V. (2019). A novel hybrid credit scoring model based on ensemble feature selection and multilayer ensemble classification. *Computational Intelligence*.

[27] Tripathi D., Edla D. R., Cheruku R. (2018). Hybrid credit scoring model using neighborhood rough set and multi-layer ensemble classification. *Journal of Intelligent & Fuzzy Systems*.

[28] Balogun A. O., Basri S., Abdulkadir S. J., Hashim A. S. (2019). Performance analysis of feature selection methods in software defect prediction: a search method approach. *Applied Sciences*.

[29] Akintola A. G., Balogun A., Lafenwa-Balogun F. B., Mojeed H. A. (2018). Comparative analysis of selected heterogeneous classifiers for software defects prediction using filter-based feature selection methods. *FUOYE Journal of Engineering and Technology*.

[30] Balogun A. O., Basri S., Mahamad S. (2020). Impact of feature selection methods on the predictive performance of software defect prediction models: an extensive empirical study. *Symmetry*.

[31] Kolukisa B., Yavuz L., Yavuz L. (2020). Coronary artery disease diagnosis using optimized adaptive ensemble machine learning algorithm. *International Journal of Bioscience, Biochemistry and Bioinformatics*.

[32] Latha C. B. C., Jeeva S. C. (2019). Improving the accuracy of prediction of heart disease risk based on ensemble classification techniques. *Informatics in Medicine Unlocked*.

[33] http://archive.ics.uci.edu/ml/datasets/statlog+(heart).

[34] Ng A. Y. Feature selection, L1 vs. L2 regularization, and rotational invariance.

[35] Farrell M. D., Mersereau R. M. (2005). On the impact of PCA dimension reduction for hyperspectral detection of difficult targets. *IEEE Geoscience and Remote Sensing Letters*.

[36] Versaci M., Angiulli G., di Barba P., Morabito F. C. (2020). Joint use of eddy current imaging and fuzzy similarities to assess the integrity of steel plates. *Open Physics*.

[37] Lee S.-H. (2015). Feature selection based on the center of gravity of BSWFMs using NEWFM. *Engineering Applications of Artificial Intelligence*.

[38] Smith M.-C., Barber P. A., Stinear C. M. (2017). The TWIST algorithm predicts time to walking independently after stroke. *Neurorehabilitation Neural Repair*.

[39] Liu X., Wang X., Su Q. (2017). A hybrid classification system for heart disease diagnosis based on the RFRS method. *Computational and Mathematical Methods in Medicine*.

[40] Jothi Prakash V., Karthikeyan N. K. (2021). Enhanced evolutionary feature selection and ensemble method for cardiovascular disease prediction. *Interdisciplinary Sciences: Computational Life Sciences*.

